# A Novel Approach to Visualize Liquid Aluminum Flow to Advance Casting Science

**DOI:** 10.3390/ma16020756

**Published:** 2023-01-12

**Authors:** Casey Bate, Philip King, Jay Sim, Guha Manogharan

**Affiliations:** Department of Mechanical Engineering, Penn State University, State College, PA 16801, USA

**Keywords:** mold filling, sand-casting, succinonitrile, casting hydrodynamics, water modeling, metal analog, flow simulation

## Abstract

Turbulent filling of molten metal in sand-casting leads to bi-films, porosity and oxide inclusions which results in poor mechanical properties and high scrap rate of sand castings. Hence, it is critical to understand the metal flow in sand-molds, i.e., casting hydrodynamics to eliminate casting defects. While multiple numerical methods have been applied to simulate this phenomenon for decades, harsh foundry environments and expensive x-ray equipment have limited experimental approaches to accurately visualize metal flow in sand molds. In this paper, a novel approach to solve this challenge is proposed using Succinonitrile (SCN) as a more accurate metal analog in place of water. SCN has a long history in solidification research due to its BCC (Body-Centered-Cubic) crystal structure and dendrite-like solidification (melting temperature ~60 °C) like molten aluminum. However, this is the first reported study on applying SCN through novel casting hydrodynamics to accurately visualize melt flow for casting studies. This paper used numerical simulations and experiments using both water and SCN to identify the critical dimensionless numbers to perform accurate metal flow analog testing. Froude’s number and wall roughness were identified as critical variables. Experimental results show that SCN flow testing was more accurate in recreating the flow profile of molten aluminum, thus validating its utility as a metal analog for metal flow research. Findings from this study can be used in future metal flow analysis such as: runner, in-gate and integrated filling-feeding-solidification studies.

## 1. Introduction

Metal casting is the oldest known manufacturing process and plays a role in 90% of all manufactured goods [1]. In particular, 80% of castings are produced via the traditional sand-casting method [2]. The traditional sand-casting process involves mold fabrication (e.g., no-bake green molds) using a pattern to produce mold components (e.g., cores, cope, drag and cheeks) that are assembled at the parting line. In addition to generating the mold cavity for part geometry, the pattern plate also develops the geometry of gating system (i.e., channels for metal flow into the mold cavity). The gating system in sand-casting includes: a pouring basin, sprue, runners, gates and risers. Several studies in traditional sand-casting have highlighted the importance of optimizing gating system design to minimize casting defects [3,4].

One of the inherent challenges in traditional sand-casting is minimizing turbulence in the melt flow such that critical velocity (<0.5 m/s) is not exceeded at the ingate [5,6]. Achieving this condition reduces air entrainment, splashing, and film formation, all of which contribute to casting defects during solidification and reduce part strength [7,8]. Turbulence could be minimized through proper design and analysis of gating systems [6]. Recent innovations in 3D sand-printing (3DSP) have enabled non-conventional gating system designs since geometric limitations of traditional mold fabrication have been greatly reduced [9]. Such 3DSP-centric gating designs can significantly reduce overall casting defects by as high as 99.5%, oxide inclusions by 35% and improve mechanical strength of metal casted parts by 8.4% that would both improve production costs for foundries by limiting scraps and improve overall part quality for end-applications [10].

Experimental analysis of liquid metal flow has been a major challenge in casting research [11,12,13]. This can be attributed to the pouring conditions in castings (e.g., opaque sand-molds, harsh environments including high temperature, outgassing, abrasive sand, humidity) that do not provide easy access to collecting qualitative or quantitative data. Consequently, computational fluid dynamics flow and solidification simulations are widely used for evaluating mold design [14,15,16]. Previous studies have collected flow field data using expensive X-ray equipment but could provide only qualitative data [17,18]. However, such an approach is restricted due to limited feasible geometries that could be analyzed through X-ray, high experimental cost, safety concerns, 2D imaging which does not capture the vortexes in metal flow and lower resolution. Several studies have reported attempts to collect temperature and deduced velocity data via in-contact thermal measurement sensors with limited success due to dynamic changes in temperatures and conductivity of the melt [19,20]. 

**Figure 1:**
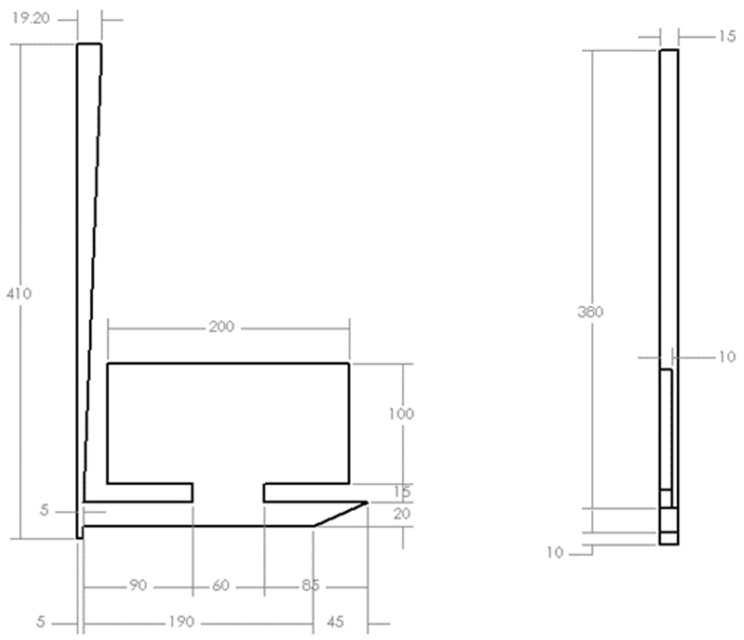
Casting geometry based on prior experimental study [17], units in mm.

In 1996, a benchmark study, motivated by the rapid growth in research efforts on numerical modeling, was conducted to characterize liquid metal flow in a sand mold to cast an aluminum benchmark plate (10 mm × 200 mm × 100 mm) [17]. As shown in Figure 1, a bottom gating system with a runner of 240 mm length and sprue height of 410 mm from entrance to sprue well was fabricated with an offset pour basin that featured a removable plug. 

After a predetermined melt height was reached in the pouring basin, the plug was removed to eliminate the effects of initial velocity during pouring. A 2.2 kg charge of 99.99% pure aluminum was poured into the basin at 720 °C (approximately 700 °C) at the sprue entry based on solidification simulation—SolidCast). The mold filling (*n =* 3) was recorded in an X-ray machine at a sampling frequency of 50 Hz. The images captured in this study are the most prevalently employed benchmark for sand mold filling studies. 

**Figure 2:**
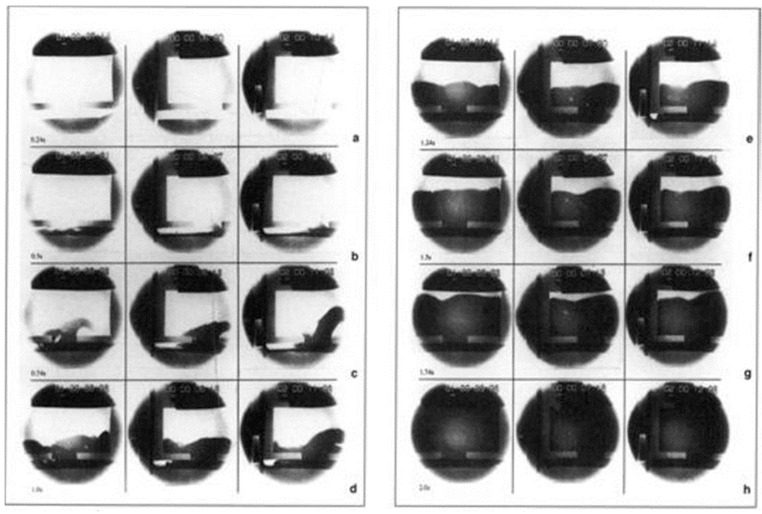
Filling images from [17] study. Each column shows 1 of 3 tests corresponding to the time from initial filling: (**a**) 0.24 s, (**b**) 0.5 s, (**c**) 0.74 s, (**d**) 1.0 s, (**e**) 1.24 s, (**f**) 1.5 s, (**g**) 1.74 s, and (**h**) 2.0 s.

The need for melt flow experiments is derived from the need to evaluate and verify existing, and ever-growing numerical models for metal flow. Pioneering work by Johnson et al. [21] classified pressurized and non-pressurized melt flow and presented the early guidelines on ingate design for liquid Aluminum. In the same era of 1950s, Richins and Wetmore experimentally showed the importance of tapered sprue that blends into the pouring basin to prevent aspirations in liquid metal during pouring [22]. In recent years, Skov-Hansen and Green employed glass fronted sand molds to compare real time x-ray flow patterns of molten ductile iron and optical imaging as shown in Figure 2 [17,23]. Tiedje experimentally showed that smaller particles produced due to splashing in the runner can freeze faster than in the case of laminar melt flow [24]. In another experimental study, Tiedje and Larsen recommended that pressure fields are more effective in elucidating melt flow stabilities and sharp changes in rigging design results in pressure waves which often leads to defective castings [25]. In recent years, Cao et al. [26] showed that in the case of high pressure die casting, fast shot velocity will greatly influence the location of the back flow junction which could result in more porosity in the die casting. 

Although these reported studies use glass fronted molds, the ability to produce freeform geometry that can be used to visualize metal flow in 3D freeform sand molds cannot be achieved due to inherent challenges in producing complex glass geometries. For instance, there is a need to visualize the melt flow in complex 3D conical helix sprue which has shown significant improvements in casting performance [10]. On the other hand, such geometries can be produced using direct 3D printing of transparent polymers or machined from acrylic (in the case of parabolic sprues). Hence, it will be desirable to evaluate alternative liquid metal analogs that can be investigated near room temperature. Water has been commonly used in the casting research community as a cheaper alternative to study liquid metal flow. Additional studies have used water to study liquid steel flow in continuous casting settings [27,28]. In both studies, water was used to verify the accuracy of a numerical model of fluid flow to indirectly develop the boundary conditions of the model intended for liquid metal [27,28]. Cleary et al. used water to model molten aluminum flow through die cavities as the control for a novel smoothed-particle hydrodynamic (SPH) model and commercially available casting flow simulation tools [11]. The study found that flow simulations had resemblance to water tests. Renukananda et al. also used water to examine mold filling in a horizontal multi-gate system and showed that while water had a different gate velocity and flowrate than the comparison metal, the relationship between these properties and gate location followed similar trends but was not accurate to estimated flow parameters [13].

The reliance on water tests as a means of numerical confirmation for liquid metals is an approximate ‘similarity testing’. Water has different thermal-fluid properties when compared to liquid metal and unlike molten liquid metal, does not solidify at room temperature. This leads to limitations in its utility as an evaluation tool that can both simultaneously and accurately represent liquid metal flow and solidification. Several studies have been conducted to determine factors that are critical to establish metal-water analogs [29,30]. These studies have primarily focused on a select group of dimensionless numbers: Froude number (Fr), Reynolds number (Re), and Weber number (We).

(1) $$\mathrm{Fr}=\frac{V^{2}}{{\mathrm{gD}}_{h}}$$

(2) Re=ρVDhμ

(3) $$\mathrm{We}=\frac{{\rho V}^{2}D_{h}}{\sigma }$$

Froude number is a ratio of inertial to gravity forces acting on a fluid, Reynolds number is a ratio of inertial to viscous forces, and Weber number is a ratio of inertial to surface tension where V, g, D_h_, ρ, µ, and σ are respectively the velocity, gravity (other forces may be considered when using centrifugal, tilt die, or high pressure die casting is used), hydraulic diameter, density, dynamic viscosity, and surface tension. Another study showed that matching Reynolds number in reduced-scale water models for continuous casting tundishes could achieve reliable metal-water analogy [29]. Froude number was determined to have no effect on these systems [29]. Another study claimed that despite a difference of about 18% between the kinematic viscosities of water and steel, limited variation was observed in the flow patterns of the two materials in the nozzle condition [27]. Another study matched Fr, Re, and We in water models to simulate air entrainment in plugging steel flows [31]. Despite these efforts, it was analyzed that air entrainment would be higher in actual steel processing based on observations in water tests. In summary, there are major unresolved issues in using water analog tests for melt flow analysis which is the motivation of this study: identify-design-evaluate alternative approaches to visualize metal flow in casting. 

Succinonitrile—C_2_H_4_(CN)_2_ has a low entropy of fusion and is a single plastic from −35 °C to 62 °C melting temperature [32]. It has been the focus of several decades of solidification research since being popularized by Glickman et al. in 1976 [33] with earlier studies into molecular and vibrational modes conducted in the mid 1950’s by Janz and Fitzgerald [34,35]. SCN’s properties as a “plastic crystal” are popular in the field of crystal growth science focused on dendritic solidification. Dendrite solidification is the process of crystal formation in metals such as nickel, copper, gold, silver, aluminum, zinc, lead, tin, and indium [36,37,38]. Plastic crystals such as SCN are a class of molecular solids (both organic and inorganic) which melt with a relatively small entropy change. Hence, they are considered as analogs to simple metals for solidification studies. The rotary motions of these molecules are preserved when a molecule transforms from liquid to solid phase. Plastic crystals typically have a wide liquid range when compared to most substances that melt closer to ambient temperature. Additionally, the transparency of plastic crystals makes it suitable for a wide variety of optical techniques for accurate morphological and kinetic measurements [33]. 

The pioneering study by Glickman drew numerous conclusions about the physics of dendrite modeling while expanding knowledge on physical properties of SCN [33]. Subsequent studies continued to employ SCN for dendrite formation studies [39,40,41,42,43,44,45]. Another study explored the addition of argon gas and acetone to SCN, and acetone with SCN in 1988 [46]. Acetone was of particular interest to form an SCN alloy that preserved the linear solid-liquidus line in the SCN phase diagram. SCN has continued to be popular in solidification research as researchers continue to focus on more specific areas of solidification and grain refinement [47,48,49]. It has also garnered interests in electronics due to its solid conductive properties [50].

Another study employed light scattering spectrometry to measure the viscosity and surface tension of liquid SCN, two properties which are vital to understanding the flow and heat transfer rate that were not previously well explored [51]. Surface tension (±2%) and viscosity measurements (±10%) for six different temperatures ranging 60 °C to 110 °C were recorded for pure SCN and can be correlated to temperature as shown in (Equations (1) and (2)) [51].

Surface tension (mPa-s) = 43.14 − 0.0823 T(4)

Viscosity (mN/m) = 4.11 − 0.0263 T(5)

The density of SCN as a function of temperature (°C) is shown in Equation (6) [52].

Density (g/cm^3^) = 1000 (1.0334 − (0.000781 × T)) (6)

Additional thermal and physical properties of SCN are listed in Table 1.

The aim of this paper is to evaluate the suitability of Succinonitrile (SCN) as an alternative to water as an analog for liquid metal flow. If successful, SCN flow tests could accurately mimic metal flow with the properties of SCN as a plastic crystal. When coupled with the dendrite solidification formation at room temperature of SCN, SCN could enable novel flow solidification visualization framework. The success of SCN as a means of mimicking metal flow will further bridge the gap between metal flow and solidification models which would positively impact related experimental efforts and lead to more accurate numerical solvers for integrated flow-solidification models. The methodology detailed in this paper also provide a roadmap for validation of innovative gating geometries.

## 2. Materials and Methods

In this study, a systematic methodology to achieve similarity values in critical fluid flow parameters (Re, Fe, We) and solidification parameters were developed for the proposed flow material (liquid SCN) for targeted metal flow (Aluminum—Table 2 [17]). 

Hydraulic diameter of a fully filled rectangular channel can be found using Equation (7).

(7) $$D_{h}=\frac{2\mathrm{ab}}{a+b}$$

Based on Equation (7), a rectangular channel of 19.2 mm × 15 mm cross-section [17] will result in a hydraulic diameter of 0.01684 m. With an average head height of 40 mm during pouring, a modified version of Bernoulli’s theorem (Equation (8)) where g and h are respectively gravity and height, found that an initial velocity of 0.886 m/s was likely to occur immediately after the plug was removed.

(8) $$V_{\mathrm{inital}}=\sqrt{2\mathrm{gh}}$$

Subsequently, Re number of 28,325 was determined for the molten aluminum at the entrance of the sprue (Equation (2)):

ReAluminum=2373×0.886×0.0168420.00125=28328

There are three different approaches to achieve a desired Re (i.e., 28,325) for any material (i.e., SCN) by varying the: (1) pour temperature to correspondingly vary the kinematic viscosity of the fluid, (2) pour velocity, and/or (3) hydraulic diameter of the channel opening. 

In this case, the kinematic viscosity of aluminum was 5.268 × 10^−7^ m^2^/s. Equations (5) and (6) result in a temperature similarity value of 137.73 °C for SCN which violates the physical properties of SCN. However, a pouring temperature of 75 °C for SCN results in a velocity of 3.969 m/s (i.e., 4.17 times that of aluminum with a hydraulic diameter of 0.07017 m) as highlighted in Table 3. 

This methodology to identify similarity values was repeated for aluminum and water and it was found that water flow at a temperature at 53.1 °C would result in Reynolds number similar to molten aluminum for the same volumetric flow conditions as highlighted in Table 4. 

Weber number is of interest to analyze air entrainment in a fluid. Since SCN solidifies near room temperature, the flow of liquid SCN, if similar to molten aluminum flow, will enable accurate analyzation of air entrainment. In other words, matching similarity values of molten SCN could provide insights into the bubble trail formation during air entrainment while pouring and solidification of aluminum. Based on properties of aluminum in Table 2, Weber number of aluminum at pouring conditions (We = 36.05) cannot be directly matched with SCN at 75 °C (We = 346.6). It is well established that many issues in metal casting stem from premature solidification. Because SCN melts at lower temperatures and solidifies at room temperature, it may be able to offer new insights to this issue as an experimental tool. Chvorinov’s rule is a formula for relating solidification time to mold parameters, geometric parameters, and thermal parameters of the melt material (Equation (9)). A no-bake sand mold for a cubic casting with 0.1-m edge consist of the properties found in Table 5.

(9) $${\mathrm{Time}}_{\mathrm{Solidification}}={\left[\frac{\rho_{l}L}{T_{m}-T_{0}}\right]}^{2}\left[\frac{\pi }{4k_{m}\rho_{m}c_{m}}\right]\left[1+{\left(\frac{c\Delta T_{s}}{L}\right)}^{2}\right]{\left[\frac{V}{A}\right]}^{2}$$

k_m_ = Thermal conductivity of mold (W/m °C)
ρ_m_ = Density of mold material (kg/m^3^)
c_m_ = Specific heat of mold (J/kg °C)
V = Volume of casting (m^3^)
A = Surface area of casting (m^2^)
ρ_l_ = Liquid density of pour material (kg/m^3^)
L = Latent heat of pour material (J/kg)
c = specific heat of pour material (J/kg °C)
T_m_ = Melting temperature of pour material (°C)
T_0_ = Ambient Temperature (°C)
ΔT_s_ = Superheat, temperature at which material is pour minus melting temperature (°C)

If these mold conditions are maintained, then the solidification time of the casting becomes solely dependent on the thermal properties of the pour material. In other words, aluminum poured at 700 °C would result in a solidification time of 517.91 s using Chvorinov’s rule for the given mold conditions (aluminum density = 2373 kg/m^3^, latent heat = 398,000 (J/kg), specific heat = 1888 J/kg °C, melt temperature = 660 °C). If cast material was changed from aluminum to Succinonitrile, it was found that an identical solidification time of 517.91 s would be obtained for a superheat of 9.445 °C (meaning pour temperature of 67.445 °C) considering the properties of SCN found in Table 1 and the mold properties in Table 5. 

This ability to match the solidification time of a common casting alloy (e.g., aluminum) for a relatively smaller super-heat highlights the potential of SCN as an experimental casting research tool. The solidification research of SCN has already been discussed, however, this novel approach shows that SCN could be used as a method of studying gating systems in metal castings. This ability to match solidification time could accurately represent the problem of premature solidification in gating systems. This allows for the experimental testing of innovative gating designs (e.g., thin-wall castings). It should be noted that Chvorinov’s rule is designed to quantify conductive heat transfer of a stationary fluid through a mold after filling has been completed. Chvorinov’s rule does not consider convective heat transfer, which play a large role in the premature solidification problem, or radiative heat transfer. 

The application of SCN for casting research would lead to lower costs, fewer resources, and possibilities to visualize and quantify flow parameters. The simplest way to accomplish this goal would be to substitute the sand mold with a commonly available material that is both transparent and compatible with SCN material. Acrylic plastic is inert to SCN, easy to obtain, machine, and assemble as well as transparent and relatively cheap. It has already been used in casting experiments most notably in 2016 [13]. Unfortunately, the thermal characteristics of acrylic do not yield themselves well to the previous solidification study. Acrylic has a thermal conductivity of 0.21, a density of 1200, and a specific heat equal to 1500 (all SI units). In the case of casting SCN, solidification time would rise to 1322.73 s which is about twice that of molten aluminum in an equivalent sand mold as detailed in Table 6. A realistic super heat could not be found for SCN and acrylic using Equation (6). Glass is a transparent material that would allow for the matching of solidification time of aluminum in a sand mold, however, it is neither cheap nor resilient to thermal shock and difficult to build custom-intricate designs. Glass has thermal conductivity, density, and specific heat respectively of 0.75, 2457.6, and 834.61 for (all SI units). This would result in a SCN solidification time of 517.91 s for a super heat of 21.44 °C (pour temperature of 79.44 °C). While higher than the sand mold, this pour temperature is still well below the 266 °C boiling temperature of SCN and will be included in future studies. 

Computer simulations (Flow3D Cast, Flow Science Inc., Santa Fe, New Mexico, Dr. Flender Holding GmbH) were conducted to analyze the effect of Reynold’s number on the flow profile. Aluminum at 700 °C was compared to water at 25 °C, and SCN for the velocity and hydraulic dimeter matched conditions. The mold geometry from the 1996 study was recreated in a CAD software as shown in Figure 1 [17]. 

**Figure 3:**
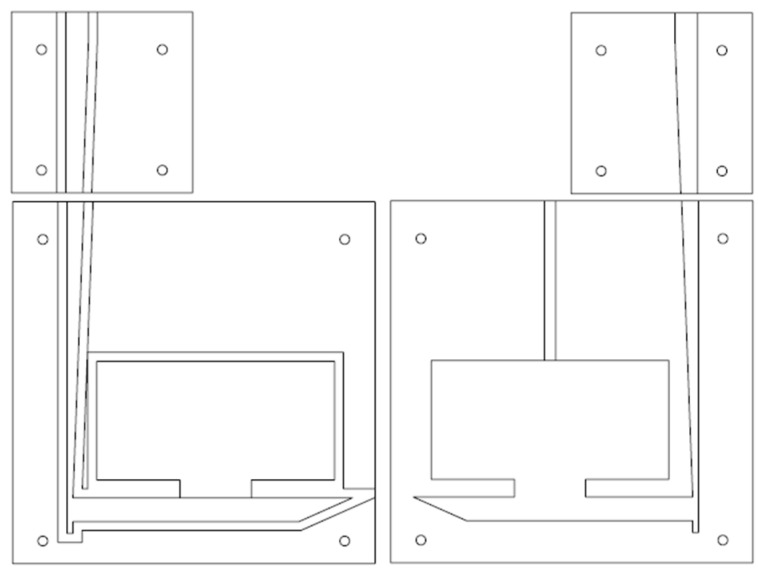
CAD design of mold parts.

Additional assumptions and limitations are listed in Table 7:

An acrylic mold was made to mimic geometry from prior study as shown in Figure 3 [17]. A CNC mill was used to cut the pattern in a 12.7 mm (0.5′′) acrylic sheets. The casting geometry was parted at the middle of the sprue so that a maximum depth of 7.5 mm (0.3) was cut into each acrylic sheet. Most of the geometry fit into a 304 × 304 mm (12′′ × 12′′) acrylic sheet. A 152 × 152 mm (6′′ × 6′′) acrylic sheet was used for the top of the sprue. A 1.3 mm (0.05′′) groove was cut around the edge of the casting geometry on one half of the mold so a rubber gasket could be applied. 

**Figure 4:**
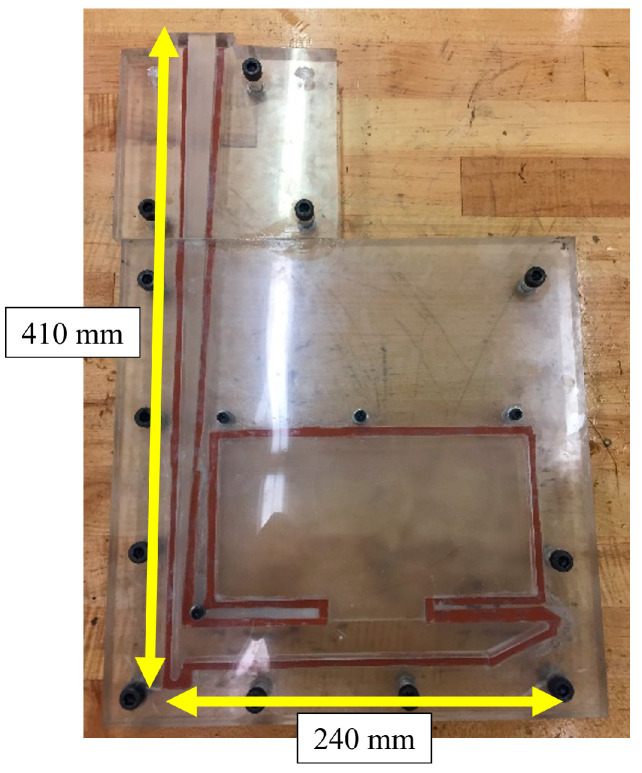
Example of assembled acrylic mold (from earlier test).

A rubber gasket of 1.6mm (1/16′′) was glued along the non-recessed edge of the gating geometry as shown in Figure 4. Clear silicon was also applied around the edges of the embedded geometry to form a seal and the acrylic parts were fastened to the glued pieces with three M8 bolts and ten 8–32 bolts. The fastening of the bolts compressed the rubber/silicon seal to form a liquid-tight seal. 

Notches were removed from the top of the sprue. A rectangular slot was cut into the bottom of a 1-quart food-grade container. The container for liquid SCN was placed on top of the mold so that the top of the sprue was aligned with the slot. The base of the container was coated with silicon to form a seal with the top of the mold. This container served as the pour basin. A rubber plug was cut to match the rectangular shape of the sprue. A screw was inserted into the plug and a string was tied around the screw to initiate pouring. Masking tape was place along the sprue and runner edges and marked every 0.5 inches so velocity data could be gathered from the mold. 

Pour testing was conducted in a fume hood due to the health hazards imposed by SCN. SCN is a category 2 skin irritant and a category 2A eye irritant [54]. For these reasons, the PPE for this work included EN 166 safety glasses, nitrile rubber gloves, type P95 respirators, and a lab coat. A Casio EXILIM high-speed camera was used to record video of the mold at 300 fps. Green food dye was added to SCN during melt in order to improve visualization against clear acrylic. The initial conditions for each tests are shown in Table 8.

A sand bath connected to a JKEM controller was used to heat the substances to the desired temperatures. For the lower temperature water tests, warm tap water was used. Each substance was poured into the pour basin until the specified head height was reached before pulling the rubber plug to initiate pouring. The compatibility and visual quality of SCN with 3D printed geometries was also included in this study. Fluid flow through 3D-printed channels have garnered much popularity and success in the biomedical field, especially for microchannel research [55,56,57,58,59]. Advances in the ability to 3D print glass structures could prove useful in overcoming the acrylic mold limitations discussed earlier [60]. For this study, a parabolic tube design was printed in a Formlab Form 1+ printer using clear resin using standard print conditions and washed in isopropyl alcohol and dried in room conditions for 24 h. After drying, the part was cured in a UV oven only for 15 min to avoid over-curing which adds a yellow hue to the print and hinders transparency. The exterior of the part was sanded with 100, 200, and 400 grade sand paper and coated with acetone. The finished part was clamped to the back wall of the fume hood. Water and SCN at 65 °C respectively were poured and analyzed using the 3D printed parabolic sprue similar to machined acrylic mold. 

## 3. Results

### 3.1. Flow Simulation Results

**Figure 5:**
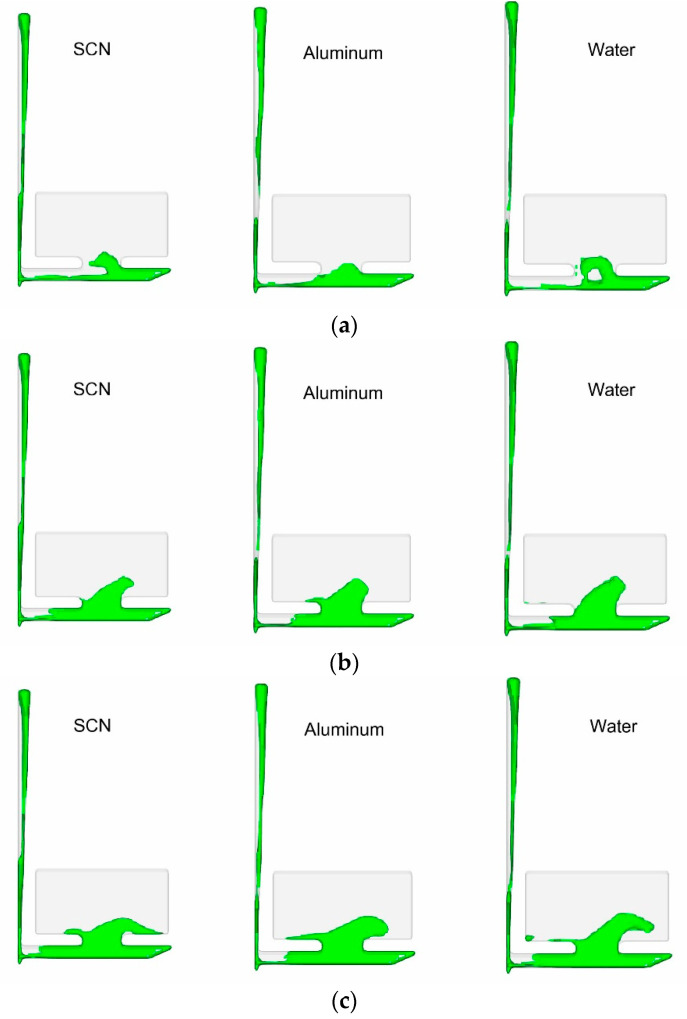

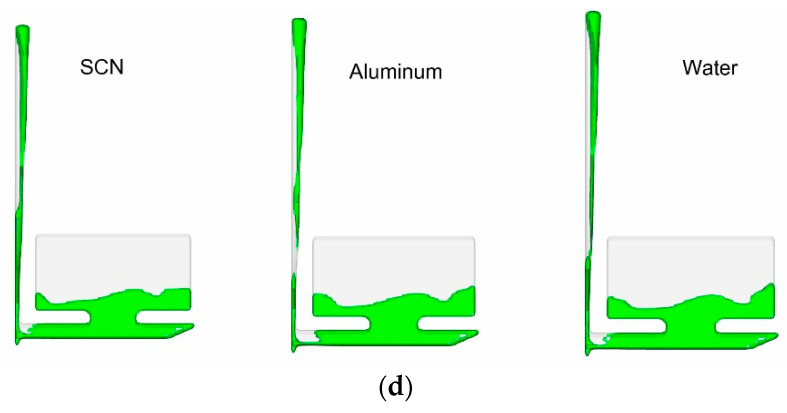
Simulation results of SCN, aluminum, and water at (**a**) 0.7s (**b**) 0.9 s (**c**) 1 s (**d**) 1.2 s.

It was found that increasing the volumetric flow rate of the SCN via greater initial velocity of larger hydraulic diameter produced different results from aluminum due to the law of continuity which states that mass flow rate of any substance must be conserved. These findings support the need for Froude number similarity in casting modeling, the need to match the mass flow rates for each material, and the limitations involved with attempting to match Reynold’s number through flowrate in casting situations. Simulation runs for aluminum at 700 °C, water at 25 °C, and SCN at 75 °C (Figure 5) showed that matching similarity values were accurate (1.2 s from the start of pour). The simulations show similar flow structures at the same times across all three materials.

### 3.2. Importance of Dimensionless Number Similarity in Water Testing

Using Bernoulli’s equation, the initial velocity and the velocity at the base of the sprue were estimated for the prior benchmark study [17]. These values were found to be 0.886 and 3.687 m/s respectively which results in an average velocity of 2.287 m/s across sprue length. 

Based on Figure 2, the sprue is filled in the first 0.24 s and the mold was fully filled in about 2 s. 

#### 3.2.1. Importance of Froude’s Number

**Figure 6:**
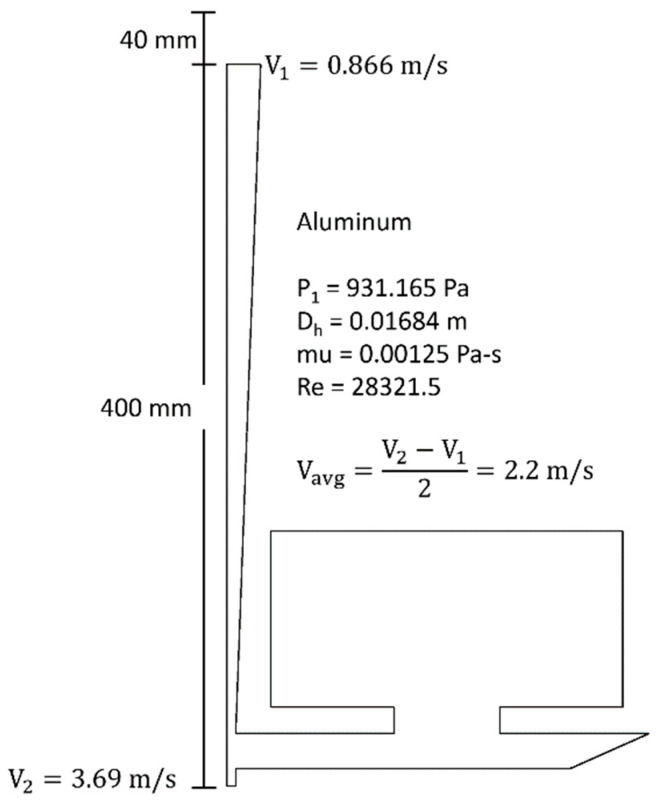
Expected aluminum flow conditions.

Mold filling time, average velocities across sprue and runner were calculated for each test. Mold filling was defined as the time at which the fluid level rose above 304 mm (12″) across the width of the plate. The average sprue velocity was calculated across fluid travel from 178 mm (7″) to 356 mm (14″) in the sprue (Figure 6). Similarly, the average runner velocity was calculated across fluid travel from 50 mm (2″) to 152 mm (6″) in the runner. These velocities represent the vertical velocity in the sprue, and horizontal velocity in the runner. As detailed in Table 9, these quantitative results showed that the higher value Froude’s number test (unmatched) exhibited higher velocities and a shorter fill time than the lower Froude’s number test. These results agree with the computational model. 

**Figure 7:**
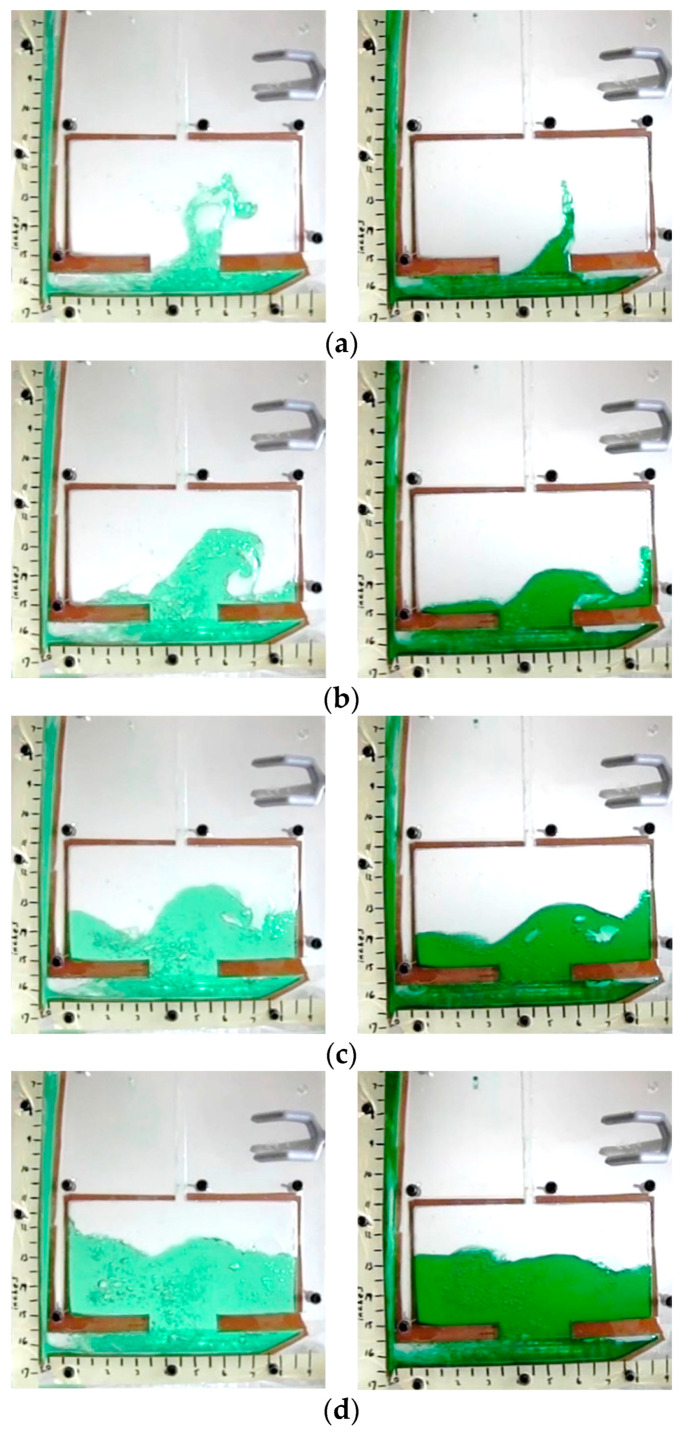
Water testing results for Fr unmatched (**left**) vs. Fr matched (**right**) (**a**) 0.5 s after pulling plug, (**b**) 0.74 s (**c**) 1 s, (**d**) 1.24 s.

Qualitative analysis also offered insight into the melt flow when compared to the aluminum casting from a prior study [17]. These images (Figure 7) show that the higher Fr value test resulted in higher kinetic energy throughout the filling for similar Reynold’s numbers. In the case of water tests, the water turbulently filled the runner as it traversed from left to right. A strong agreement between the studies was observed.

#### 3.2.2. Importance of Reynold’s Number

**Figure 8:**
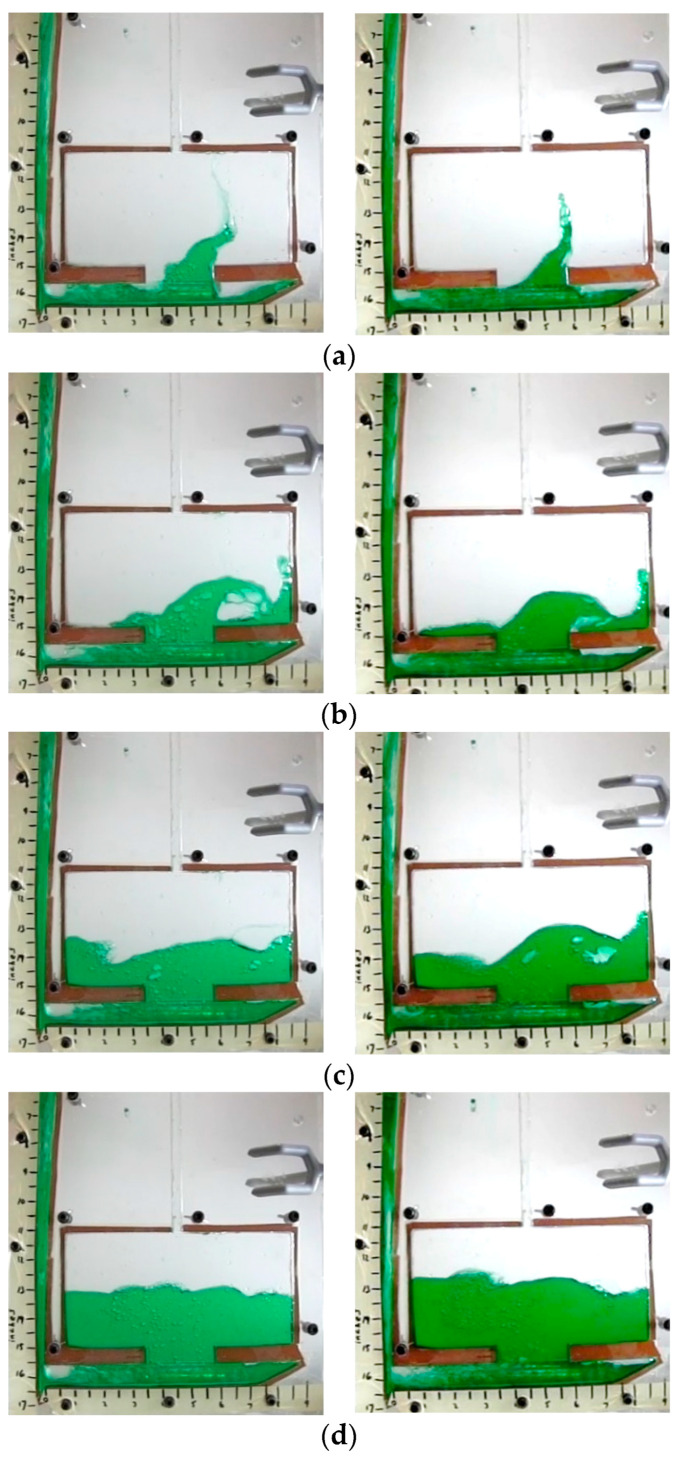
Water testing results for Re unmatched (**left**) vs. Re matched (**right**) (**a**) 0.5 s after pulling plug, (**b**) 0.74 s (**c**) 1 s, (**d**) 1.24 s.

Higher levels of agreement were observed in water tests at different Reynold’s numbers. The tests differed in filling time by 0.05 s and featured identical average sprue velocities. These results align with the computational model and Bernoulli’s equation. The qualitative analysis also shows good agreement between the two studies (Figure 8 and Table 10). 

### 3.3. Succinonitrile Comparison to Water and Aluminum

**Figure 9:**
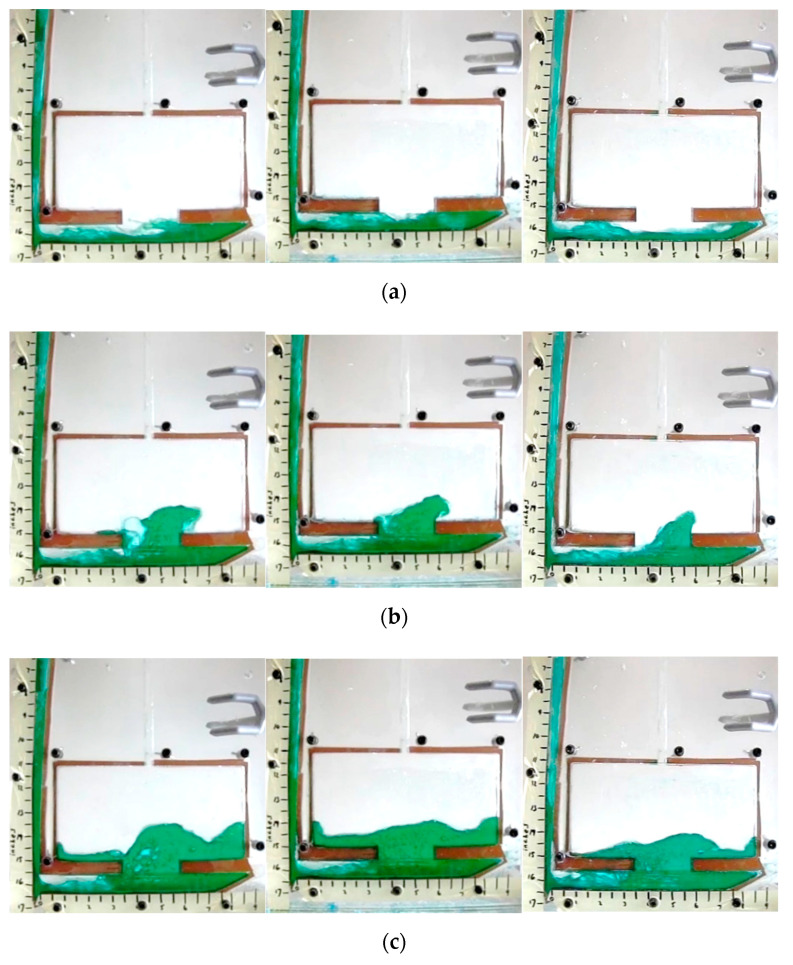

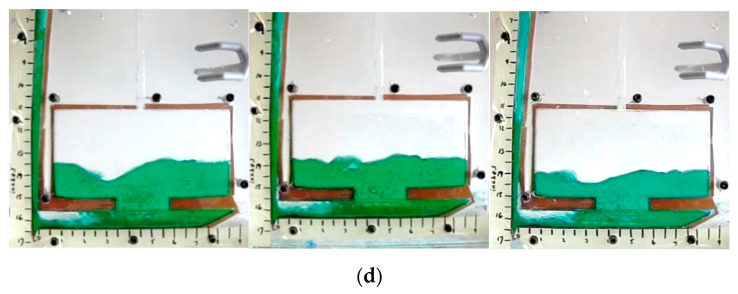
Succinonitrile testing results (**a**) 0.5 s after pulling plug, (**b**) 0.74 s (**c**) 1 s, (**d**) 1.24 s.

Qualitative results of SCN showed stronger agreement with molten aluminum tests [17] than any of the water tests. Specifically, at the 0.5 s frame, it was observed that SCN traveled across the runner in the same manner as the aluminum, and entered the plate area only after rebounding off the end of the runner. SCN entered the plate (Figure 9b) showed similar profile of molten aluminum [17]. 

SCN had a slower filling time than both water and molten aluminum despite having a faster sprue and runner velocity than water as shown in Table 11.

### 3.4. SCN and 3D Print Proof of Concept

**Figure 10:**
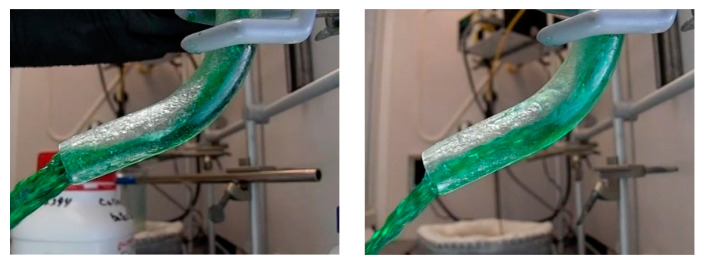
Water (**left**) and SCN (**right**) in SLA printed channel.

Both water and SCN were able to flow through the 3D printed tube without leaking or chemical incompatibility which was recorded using a Casio EXILIM high-speed camera as shown in Figure 10.

## 4. Discussion

**Figure 11:**
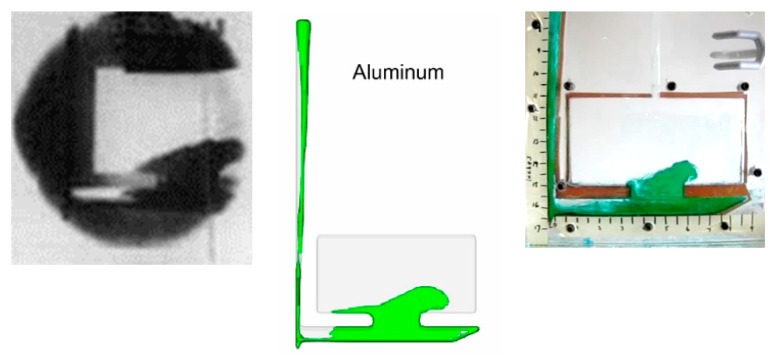
Aluminum in sand mold (**left** [17]), aluminum in sand mold simulation (**middle**) and matched SCN visualization (**right**).

This paper analyzed the ability of water and Succinonitrile (SCN) to mimic aluminum melt flow in sand casting. Matching of Reynold’s number and Froude’s number between test results concluded that it is feasible to match the casting hydrodynamics of molten metal and SCN. Figure 11 (0.74 s from start of pour) shows a simulation recreation of the aluminum pour for the same time step. 

**Figure 12:**
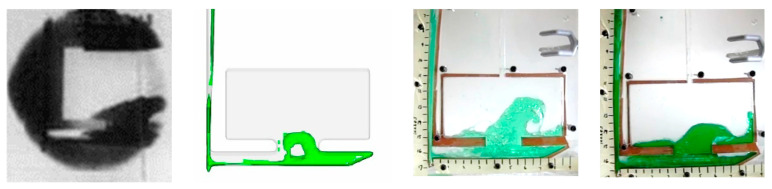
Aluminum in sand mold (**left**), Water in sand mold simulation (**left middle**), unmatched Froude’s number water in acrylic (**right middle**), Matched Froude’s number water in acrylic (**right**).

The unmatched Froude’s number tests for water used an initial head height that was double that of the aluminum. This resulted in greater turbulence in water flow compared to aluminum (Figure 12). Similarly, simulation of water flow trailed both experiments when compared to molten metal. 

On the other hand, matched Froude’s number tests used the same head height of molten aluminum [17] as shown in Figure 12. 

**Figure 13:**
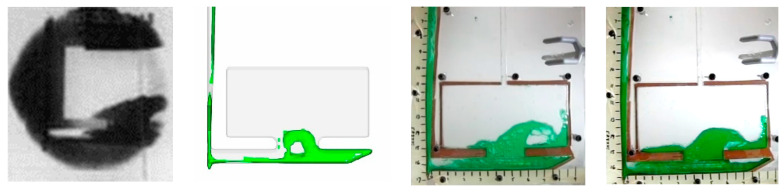
Aluminum in sand mold (**left**), Water in sand mold simulation (**left middle**), Unmatched Reynold’s number water in acrylic (**right middle**), Matched Reynold’s number water in acrylic (**right**).

The unmatched Reynold’s number test was poured at room temperature (22 °C) similar to multiple water studies reported in the literature. However, the resulting flow geometry was not similar to molten aluminum (Figure 13). 

This study hypothesized that Reynold’s number similarity between a metal and nonmetal was necessary to producing similar flow patterns. The unmatched (Figure 13) vs. matched (Figure 13) results showed minimal differences. Both images had the same degree of accuracy when compared to the aluminum pour. This finding dispels the need for Reynold’s number similarity. 

**Figure 14:**
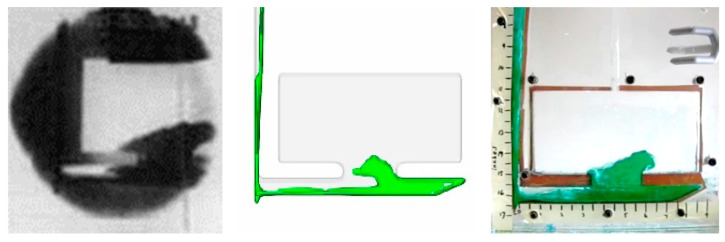
Aluminum in sand mold (**left**), Succinonitrile in sand mold simulation (**middle**), Succinonitrile in acrylic (**right**).

SCN was poured with the same Froude’s number as aluminum. The Reynold’s number of SCN was 6800, less than 25% of the aluminum (28,000). However, water tests showed that Froude’s number is significant when compared to Reynold’s number. However, the water tests used a closer range of Reynold’s numbers. The SCN results produced an accurate recreation of the aluminum pour. The fluid flow exhibited the same geometry at the same time step (Figure 14). SCN results produced a higher level of similarity to the aluminum than any of the water tests. In addition (Figure 2), only matched SCN tests and none of the water tests could recreate the flow of molten aluminum. It should be noted that although the absolute difference in time to fill across matched and unmatched conditions are in the orders of 1–5%, the resulting effects on average runner velocity varies substantially (20–80%).

**Figure 15:**
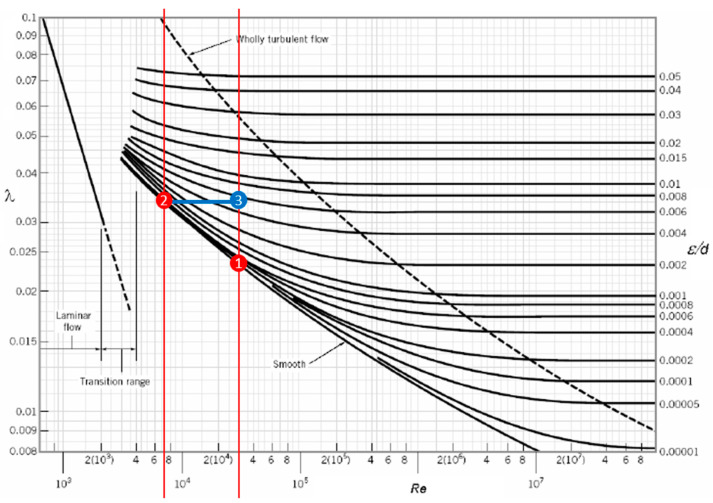
Moody diagram [61] (1) Water Re ≈ >28,000 in acrylic (2) SCN Re ≈ 6800 in acrylic (3) Relative roughness of substance at Re ≈ 28,000 correlated to pressure drop incurred by SCN.

In summary, further insight is required to explain the close similarity between the aluminum and SCN tests despite the significant differences in Reynold’s number. Insights were obtained through the Moody diagram shown in Figure 15, which relates Darcy-Weisbach friction factor to Reynold’s number. Molten aluminum (Re ≈ 28,000) and water tests were matched in Moody diagram (Figure 15). However, the relative surface roughness was different. Both water and SCN tests occurred in polished machined acrylic, which was assumed to be “smooth”. The aluminum tests occurred in a sand mold which had a higher relative roughness. This difference in surface roughness created a larger pressure drop in the aluminum, altering its flow geometry. “Smooth” walls express a near constant liner decrease in pressure drop as Reynold’s number increases. SCN tests occurred at a lower Reynold’s number which exhibited a larger pressure drop. The pressure drop during SCN testing correlated to a relative higher surface roughness value which is roughly 6 times larger than that of water. It can be concluded that pressure drop due to friction is vital to producing flow similarity between two different fluids. It should be noted that due to health and safety concerns associated with handling SCN, experiments should be conducted with appropriate use of PPE (personal protective equipment) under fume hood. While this study showed similarity between the flow characteristics of the molten Al and SCN, future work will focus on studying alternative liquid that can be used at near room temperature with less stringent PPE requirements. Finally, this study focused only on the flow similarities and did not explore the combined effects of flow conditions and resulting solidification behavior. In addition, future work should explore analogous to liquid metal that forms oxides on the surface to more accurately emulate oxide bifilms that occur commonly in melt flow.

## 5. Conclusions

To the best of authors’ knowledge, this is the first reported study on matching dimensionless casting hydrodynamics number of molten metal to SCN and water. Specifically, similarity in water and SCN flow testing for metal casting were conducted in this study and the following conclusions were deduced:

- Froude’s number similarity is important in mold filling testing in order to preserve the fill rate and total energy in fluid flow.
- Reynold’s number was not found to have a direct result on the fluid profile and pressure drop as a function of wall roughness could be a major aspect, and will be the focus of future studies.
- Water was able to roughly mimic the aluminum test, but an exact match was not achievable regardless of Reynold’s and Froude’s number similarity.
- Succinonitrile (SCN) could mimic molten aluminum tests better than water tests conducted in this study due to lower Reynold’s number at which the SCN tests and higher surface roughness of sand mold when compared to the acrylic.

This paper proposed an argument for Succinonitrile (SCN) as a better metal analog than water. The results of this study found that SCN was able to mimic the flow pattern of aluminum when compared to water. Advantages, limitations and future study of this study include:

- The ability to visualize experimental casting flow using inexpensive set-up.
- The ability to visualize experimental casting research in a safe lab setting as opposed to a foundry setting.
- The ability to rapidly create and test various gating geometries.
- The ability to reuse the test mold multiple times.
- The ability to quantify flow velocity through high-speed video imaging.
- The health hazards associated with SCN is best suited for fume hood.
- The limited thermal capacity of acrylic molds prohibited pouring SCN at higher temperatures which could lead to severe cracking of the mold and alternative material to acrylic is a focus of future studies.
- The material properties of the acrylic lead to a long solidification time for the SCN (~40 min) which limit its ability to match solidification time to that found in sand casting.

The ultimate goal of this study is to advance the manufacturing science of casting in three areas: flow visualization and quantification, advanced gating geometries, and experimental flow. The limiting factor to these advancements is the acrylic mold. Advanced material that allows for transparency, thermal capacity, and engineered surface roughness should be considered in future testing. Finally, incorporating capacitive sensors into the mold may provide a reference for detailed velocity of metal flow in traditional sand molds [62]. 

## Figures and Tables

**Table 1:** Properties of Succinonitrile (SCN).

Symbol	Property	Value	Reference
W	Molecular weight	80.092 g/mol	[33]
ΔV_m_	Molar volume change on melting	3.71 cm^3^	[33]
ρ_s_	Density of solid	1016 kg/m^3^	[33]
ρ_l_	Density of liquid	970 kg/m^3^	[33]
T_m_	Melting point	58.09 °C	[33]
T_b_	Boiling point	265.55 °C	[53]
L	Latent heat of fusion	46,238.7 J/kg	[33]
C_p_	Heat capacity of liquid	1998.23 J/kg °C	[33]
K_s_	Thermal conductivity of solid	0.224 W/m °C	[33]
K_l_	Thermal conductivity of liquid	0.223 W/m °C	[33]

**Table 2:** Properties of Aluminum.

Symbol	Property	Value
ρ_l_	Density of liquid	2373 kg/m^3^
T_m_	Melting point	640 °C
T_p_	Pouring Temperature	700 °C
μ	Dynamic viscosity	0.00125 Pa-s
L	Latent heat of fusion	398,000 J/kg
C_p_	Heat capacity of liquid	1888 J/kg °C

**Table 3:** Similarity values for Reynolds number—SCN and aluminum.

Pour Material	Aluminum	SCN	SCN	SCN
Liquid Density	2373 kg/m^3^	955.3 kg/m^3^	975 kg/m^3^	975 kg/m^3^
Dynamic Viscosity	0.00125 Pa-s	0.00051 Pa-s	0.00214 Pa-s	0.00214 Pa-s
Velocity	0.886 m/s	0.886 m/s	3.969 m/s	0.886 m/s
Hydraulic diameter	0.01684 m	0.01684 m	0.01684 m	0.07017 m
Temperature	700 °C	137.73 °C	75 °C	75 °C
Re Number	28,328	28,328	28,328	28,328

**Table 4:** Matching Reynold’s number values for water and aluminum.

Pour Material	Aluminum	Water	Water
Liquid Density	2373 kg/m^3^	997.05 kg/m^3^	986.61 kg/m^3^
Dynamic Viscosity	0.00125 Pa-s	0.00089 Pa-s	0.00052 Pa-s
Velocity	0.886 m/s	0.886 m/s	0.886 m/s
Hydraulic diameter	0.01684 m	0.01684 m	0.01684 m
Temperature	700 °C	25 °C	53.1 °C
Re Number	28,328	16,732	28,319

**Table 5:** Thermal properties of sand mold.

Property	Value
Thermal Conductivity	0.59 W/m °C
Density	1521.71 kg/m^3^
Specific Heat Capacity	1075.288 J/kg °C
Volume	0.001 m^3^
Surface Area	0.06 m^2^

**Table 6:** Solidification times for different mold materials and superheats.

Pour Material	Aluminum	SCN	SCN	SCN
Mold Material	Sand	Sand	Acrylic	Glass
Super Heat	40 °C	9.444 °C	9.444 °C	21.438 °C
Solidification Time	517.91 s	517.91 s	1322.73	517.91 s

**Table 7:** Experimental set-up assumptions.

Assumption	Explanation
Pressure head at the pouring basin is not constant.	Prior experimental study maintained a constant head height of 40 mm in the pour basis throughout the mold filling [17]. In this study, the pouring basin is filled to the required head height, and released into the mold and potential effects on velocity are neglected.
Mold permeability in acrylic molds could be accommodated for sand-molds	Mold permeability could eliminate back pressure in the mold due to trapped air. Back pressure impedes the flow of the melt. Prior study used 60 AFS-grade silica sand bonded with 1.2 wt.% phenolic urethane resin [17]. A vent was added to the test mold in this study to alleviate the back pressure.
Wall roughness was not matched.	Wall roughness plays a role in flow velocity and profile. The roughness values from prior study on sand mold [17] and that of acrylic mold in this study could differ.
Reynold’s similarity for SCN and Aluminum could be achieved at lower temperature.	Velocity and hydraulic diameter similarity were discussed in Section 2. The necessary temperature of 138 °C to thermally match exceeded the 77 °C temperature rating or the acrylic. Severe cracking was seen in attempts to reach higher temperatures. Therefore, SCN was tested at 75 °C.

**Table 8:** Testing initial conditions.

Test	Substance	Head Height	Temperature	Initial Reynolds Number
Fr matched	Water	40 mm	53 °C	28,316
Fr unmatched	Water	80 mm	34 °C	28,575
Re matched	Water	40 mm	53 °C	28,316
Re unmatched	Water	40 mm	22 °C	15,616
SCN 1	Succinonitrile	40 mm	75 °C	6804
SCN 2	Succinonitrile	40 mm	75 °C	6804
SCN 3	Succinonitrile	40 mm	75 °C	6804

**Table 9:** Froude’s number match vs unmatched.

Test	Time to Fill (s)	Average Sprue Velocity (m/s)	Average Runner Velocity (m/s)
Fr matched	1.690	1.976	1.404
Fr unmatched	1.513	2.143	2.425

**Table 10:** Reynold’s number match vs unmatched.

Test	Time to Fill (s)	Average Sprue Velocity (m/s)	Average Runner Velocity (m/s)
Re matched	1.690	1.976	1.404
Re mismatched	1.743	1.976	1.482

**Table 11:** Succinonitrile filling results.

Test	Time to Fill (s)	Average Sprue Velocity (m/s)	Average Runner Velocity (m/s)
SCN 1	2.037	2.134	1.778
SCN 2	2.107	2.134	2.319
SCN 3	2.353	2.134	1.524
